# Case Report: *Fusarium falciforme* pericardial and sternal wound infection following orthotopic heart transplantation

**DOI:** 10.3389/fcvm.2024.1480392

**Published:** 2025-01-06

**Authors:** Jeffrey Rodgers, Morgan Hill, Sanford Zeigler

**Affiliations:** ^1^ College of Medicine, Medical University of South Carolina, Charleston, SC, United States; ^2^ Division of Cardiothoracic Surgery, Department of Surgery, Medical University of South Carolina, Charleston, SC, United States

**Keywords:** heart transplant, fungal infection, mediastinitis, infection, transplant, wound vac, wound vac therapy

## Abstract

*Fusarium*, a genus of soil and vegetation-based fungi, is a rare cause of infections in immunocompromised individuals, including transplant recipients. In this case, we describe successful treatment of *Fusarium falciforme* mediastinitis in the recipient of an orthotopic heart transplant. Treatment included multiple courses of combination antibiotic and antifungal therapy several surgical debridements, continuous mediastinal irrigation with antifungal agents, and staged closure with an omental flap. This is the first report describing successful eradication of *fusarium sp.* mediastinitis and provides a template for treating complex cases of mediastinitis and osteomyelitis.

## Introduction

*Fusarium* species are soil and vegetation-based fungi that can cause infections in immunocompromised individuals (1). This includes recent solid organ transplant recipients who are placed on immunosuppression, posing a unique challenge in postoperative management (1). Once diagnosed, invasive fungal infections are associated with an approximately 60% overall case-fatality rate among solid organ transplant recipients (2). Here, we present a case of successfully treated pericarditis and osteomyelitis caused by *Fusarium falciforme* in a patient following orthotopic heart transplant. To our knowledge, this is the first reported case in which sternal and pericardial infection with this organism was successfully treated.

## Case description

A 54-year-old male with end-stage heart failure with a past medical history of type 2 diabetes (HbA1c 6.5), sarcoidosis with cardiomyopathy, ventricular tachycardia, and hypertension presented to our institution with worsening fatigue secondary to exacerbation of preexisting heart failure. Prior interventions included cardiac resynchronization therapy with cardioverter defibrillator implantation. Five days after admission, he was transferred to the cardiac ICU due to a decline in hemodynamic status. Inotropic therapy was initiated, and his transplant listing status was upgraded to 1A, meaning he was highest priority for receiving a donor heart.

Six weeks after admission, during which time the patient underwent optimization with milrinone and diuretic therapy, a donor heart became available. Bacterial culture from the donor heart did not grow any microorganisms. During the perioperative course, the patient was treated with nystatin 500,000 units mouthwash four times per day and fluconazole 200 mg IV every 48 h. Post-operatively, the patient was placed on an immunosuppressive regimen including mycophenolate, tacrolimus, and methylprednisolone.

On postoperative day (POD) 10, he developed significant leukocytosis and was treated empirically with broad spectrum antibiotics and micafungin 100 mg IV once daily for 3 days. On POD 11, the patient was found to have a large pericardial effusion requiring pericardial window and drainage. Cultures of the pericardial fluid grew *Fusarium falciforme*, as identified through combined phenotypic characterization and DNA sequencing.

In collaboration with the Infectious Disease service, the patient was started on voriconazole 350 mg IV twice daily and amphotericin B 650 mg IV daily. Two weeks following these recommendations, the strain of *Fusarium* was determined to be resistant to voriconazole. Amphotericin B was added for its suggested synergy with voriconazole (3). Prolonged use of amphotericin B led to acute kidney injury requiring intermittent dialysis. This ultimately progressed to chronic renal failure.

With this treatment, blood cultures remained negative. Five weeks after transplantation, the patient was discharged on IV amphotericin B 650 mg daily monotherapy through a peripherally-inserted central catheter (PICC). Immune suppression consisted of oral mycophenolate 500 mg four times daily and tacrolimus 0.5 mg twice daily.

**Figure 1:**
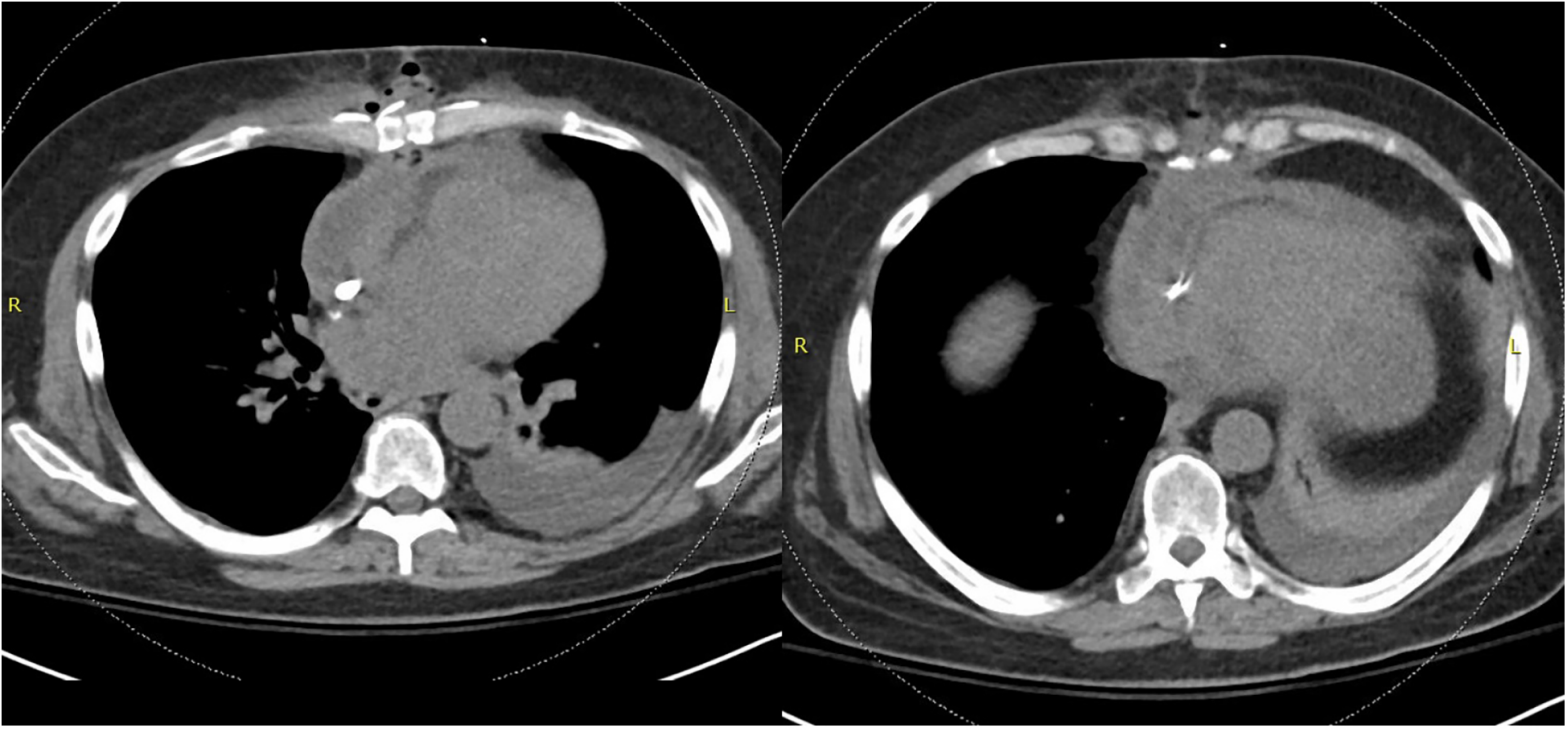
Non-contrasted CT chest demonstrating dehiscence of the chest wall incision with inflammatory changes and gas concerning for deep sternal wound infection.

Six days after discharge, the patient was readmitted with nausea, vomiting, and fever. Admission labs were notable for leukocytosis and imaging revealed dehiscence of the chest wall incision with inflammatory changes and gas surrounding the sternotomy concerning for deep sternal wound infection and osteomyelitis (Figure 1). The patient was promptly restarted on IV voriconazole 500 mg every 12 h twice as a loading dose followed by 300 mg every 12 h for maintenance dosing.

The patient was taken to the operating room urgently. Loculated, turbid, foul-smelling fluid was encountered in the pericardial space. All soft loculations were debrided to completely mobilize the heart. Superficial soft tissue and the sternal edges were debrided with curets. Osteomyelitis appeared limited. The cavity was then irrigated with voriconazole irrigation followed by pulse lavage with voriconazole irrigation.

**Figure 2:**
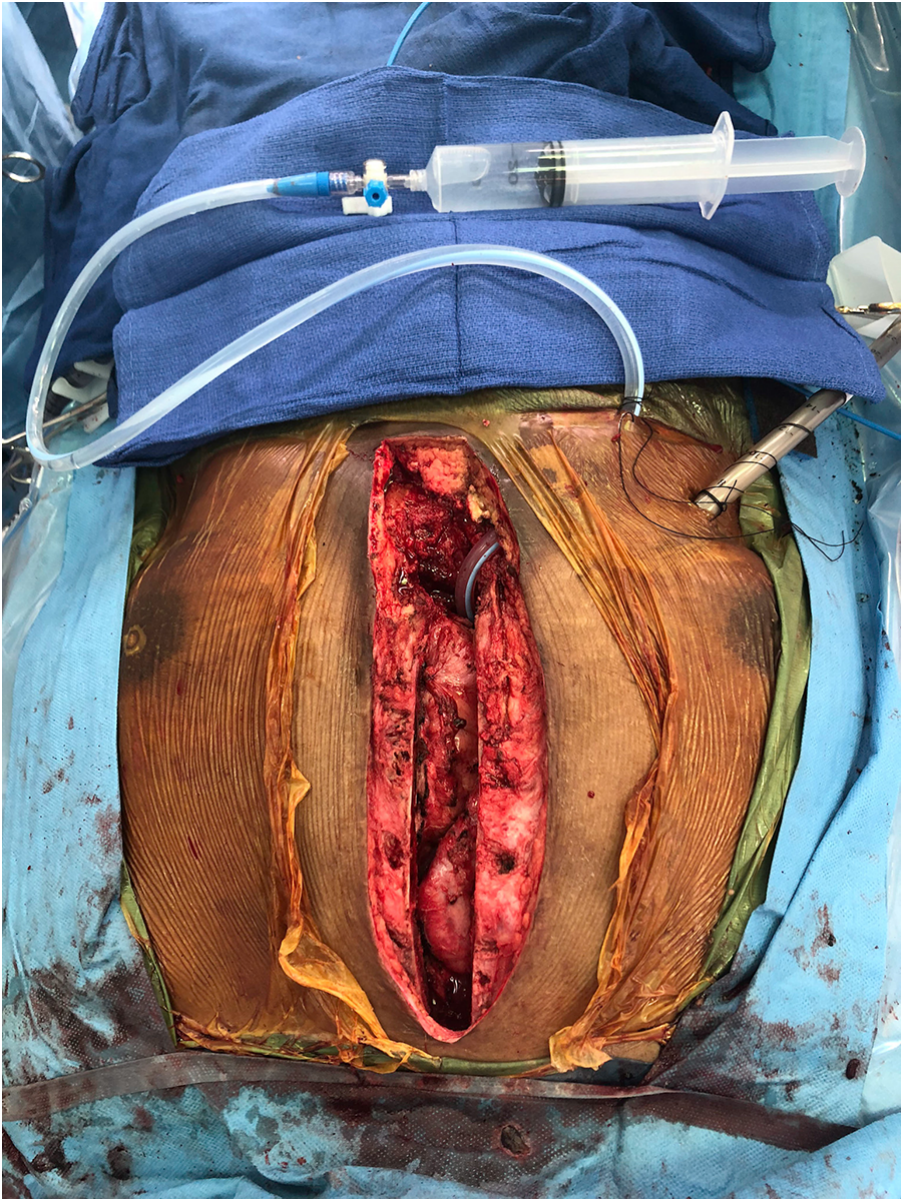
An example setup of the continuous wound irrigation system. A 19 Fr Blake drain is placed in the posterior pericardium and tunneled through the subcutaneous tissue before being secured in the usual manner. This setup is proceeded by the placement of wound vacuum sponges in the anterior mediastinum, followed by sternal closure. Voriconazole solution is flushed through the drain.

A continuous irrigation system and negative pressure therapy system was then constructed, as demonstrated in Figure 2. This was designed to allow antimicrobial solution to percolate from the posterior mediastinum anteriorly through the sternotomy and out the dressing. A 19 Fr Blake drain was placed in the posterior pericardium, tunneling it through the subcutaneous tissue and securing it in the usual manner. The wound was irrigated with voriconazole solution through the drain. Negative pressure wound vacuum sponges were then placed in the anterior mediastinum and between the sternal edges. The wound vacuum was initially irrigated every 12 h with amphotericin B solution. However, later postoperative cultures from this initial debridement grew methicillin-resistant *Staphylococcus aureus* (MRSA). This prompted the alternation of Adam's solution containing bacitracin, vancomycin, and gentamicin in addition to amphotericin B for the known *Fusarium* infection. Two consecutive debridements were performed three and four days after admission, respectively. During each of these two washouts, the mediastinum had no evidence of purulence or uncontrolled infection. Posterior irrigation and anterior vacuum suction was continued.

We then planned for definitive closure using an omental flap. Eleven days after admission, the patient underwent laparoscopic omental flap harvest. The sternal wound vac was removed and the wound was explored and debrided. The flap was then passed through a cruciate incision, two fingerbreadths in width, in the diaphragm and tunneled into the pericardial space. Bilateral pectoralis advancement flaps were then harvested. The omental flap was tacked to cover the aorta and right atrium. 19 Fr Blake drains were placed beneath each myocutaneous pectoralis flap, and the flaps were then advanced to the midline and closed over the omental flap. The superficial closure was buttressed with retention sutures. The sternum was left in non-union. An incisional vacuum dressing was placed.

The patient was treated with 8 weeks of IV vancomycin for MRSA mediastinitis and bacteremia, and 6 months IV amphotericin B for fungal mediastinitis. His renal failure persisted and progressed to ESRD. He required a PEG tube for nutrition. Eight weeks from his admission, the patient was discharged to a subacute rehabilitation facility with a PICC line in place. Blood cultures remained negative. Beta-D glucan levels remained elevated for 6 months after his re-exploration and were normal 18 months postoperatively.

The patient survived 5 years since heart transplant. He remained without severe primary graft dysfunction with an ejection fraction >55% without RV or LV dysfunction. He had no recurrence of bloodstream infection until he died. At that time, he was preparing to be listed for kidney transplantation, and developed recurrent MRSA bacteremia related to his dialysis access.

## Discussion

*Fusarium falciforme* is a ubiquitous species of *Fusarium* that exists in the air, soil, water, and vegetation (1). Localized infections can occur in both immunocompetent and immunocompromised individuals, and normally first noticed by a dermatologic presentation (1, 4). They can also be diagnosed radiographically with CT imaging or 18-FDG PET/CT imaging. *Fusarium* has also been shown to be a rare agent of disseminated infection in immunocompromised individuals, which has been reported in patients in treatment for acute myeloid leukemia and other individuals with profound neutropenia (1, 5). However, fusariosis is only rarely associated with recent solid organ transplantation, including orthotopic heart transplantation (4). This patient developed a localized fusarium infection that was also associated with MRSA wound infection and bacteremia.

Data regarding the treatment of disseminated and visceral *Fusarium* infection is somewhat lacking because of the low occurrence of disseminated *Fusarium* infections with successful treatment. Previous studies suggest current first-line treatment for disseminated fusariosis with either voriconazole, amphotericin B, or liposomal amphotericin B (4). However, other studies have noted the potential limitation of using amphotericin B monotherapy in the eradication of this fungal agent (6). Studies looking at combination therapy for this disease is even more limited (1). For this unique case, it was decided to treat his fungal pericarditis and osteomyelitis with a combination of amphotericin B and voriconazole, as this had been reported to be previously successful in a case of *Fusarium* infection causing endocarditis (7). Though sensitivity studies determined that this strain of *Fusarium* had resistance to voriconazole alone, it was continued as part of the therapeutic regimen because of the previously reported synergy among amphotericin B- and voriconazole-based therapies, respectively (3).

To our knowledge, this is the first report of a continuous irrigation system containing an antifungal solution for a patient with fungal mediastinitis. Previous studies have reported thoracic continuous saline irrigation systems have been constructed for the purpose of descending necrotizing mediastinitis (8). In this study, nonantibiotic saline irrigation was constructed in addition to standard-of-care IV antibiotics. Even so, continuous saline irrigation resulted in significantly decreased length of hospital stay and a significantly shorter drainage period than patients with necrotizing mediastinitis who received the current standard of care. Another previous case report demonstrates the efficacy of continuous antifungal irrigation in a case of renal infection (9). In our patient, we decided to apply this continuous irrigation to fungal mediastinitis by adding voriconazole and later, amphotericin B to the saline irrigation solution.

Another unique aspect of the treatment of our case was the use of an omental flap for the use of fungal mediastinitis. This two-step omental flap creation was described previously for the successful resolution of Candidal aortic graft infection and omental flaps have been used in a variety of thoracic defects, including sternal wound infections, bronchopleural fistulas, chest wall defects, and other reconstructive operations (10). As the authors noted, an omental flap absorbs infectious secretions while also providing a surface of increased vascularization for antibiotic delivery (10). Similarly to the patient described in the previous case, this mediastinal omental flap allowed for recovery after the continuous antifungal irrigation solution.

## Conclusions

In conclusion, here we describe a unique case of an orthotopic heart transplant complicated by mediastinal *Fusarium falciforme*. The patient was successfully treated with a combination of antibiotic therapy, surgical debridements with continuous antifungal irrigation system installation, and eventual closure with mediastinal omental flap insertion. Overall, we demonstrate the successful combination of multiple treatment modalities in individuals with rare post-transplantation fungal mediastinal infections.

## Data Availability

The raw data supporting the conclusions of this article will be made available by the authors, without undue reservation.
